# Nanotechnology: An Untapped Resource for Food Packaging

**DOI:** 10.3389/fmicb.2017.01735

**Published:** 2017-09-12

**Authors:** Chetan Sharma, Romika Dhiman, Namita Rokana, Harsh Panwar

**Affiliations:** ^1^Department of Dairy Microbiology, College of Dairy Science and Technology, Guru Angad Dev Veterinary and Animal Sciences University Ludhiana, India; ^2^Department of Microbiology, D.A.V. College for Girls Yamuna Nagar, India

**Keywords:** nanotechnology, packaging, nanoparticles, antimicrobial, nanosensors

## Abstract

Food commodities are packaged and hygienically transported to protect and preserve them from any un-acceptable alteration in quality, before reaching the end-consumer. Food packaging continues to evolve along-with the innovations in material science and technology, as well as in light of consumer's demand. Presently, the modern consumers of competitive economies demands for food with natural quality, assured safety, minimal processing, extended shelf-life and ready-to-eat concept. Innovative packaging systems, not only ascertains transit preservation and effective distribution, but also facilitates communication at the consumer levels. The technological advances in the domain of food packaging in twenty-first century are mainly chaired by nanotechnology, the science of nano-materials. Nanotechnology manipulates and creates nanometer scale materials, of commercial and scientific relevance. Introduction of nanotechnology in food packaging sector has significantly addressed the food quality, safety and stability concerns. Besides, nanotechnology based packaging intimate's consumers about the real time quality of food product. Additionally, nanotechnology has been explored for controlled release of preservatives/antimicrobials, extending the product shelf life within the package. The promising reports for nanotechnology interventions in food packaging have established this as an independent priority research area. Nanoparticles based food packages offer improved barrier and mechanical properties, along with food preservation and have gained welcoming response from market and end users. In contrary, recent advances and up-liftment in this area have raised various ethical, environmental and safety concerns. Policies and regulation regarding nanoparticles incorporation in food packaging are being reviewed. This review presents the existing knowledge, recent advances, concerns and future applications of nanotechnology in food packaging sector.

## Introduction

Global food industry is under rising pressure to meet consumers demand for safe, healthy and fresh food, along with a challenge to meet updated strict food safety regulations. Awareness through easy access to digital media have motivated consumer toward demand for fresh, minimally processed, nutritious, safe and ready-to-eat food products with well-defined labels. In-order to make certain the safety and authenticity of food stuffs throughout the food supply chain, food manufacturers, traders, buyers and food regulatory authorities seeks a novel, cost-effective, fast and consistent tool to monitor the packaged food quality; more effectively to the traditional passive barrier concept, as packaging is a crucial component of every segment of food industry (Janjarasskul and Suppakul, 2016; Sarkar et al., 2017). Nano-technological interventions to food packaging chiefly explore three possibilities *viz*. direct incorporation into food products, incorporation in food packaging material, and application in food processing. The commercialization, successful execution and responses to diverse applications of nanotechnology are determined by the consumer's outlook toward and acceptance of newly introduced technologies and their applications (Gupta et al., 2011; Kim et al., 2014). Outcome from majority of the studies (apart from those of sample population) indicates wide consumer acceptance of nanoparticles as packaging materials and also when they are used during processing activities; as compared to their direct incorporation into agri-food products (Giles et al., 2015).

The concept of nanotechnology was introduced in 1959 by Richard Feynman and the term “nanotechnology” was later coined by Norio Taniguchi in 1974. Nanotechnology mainly comprises of fabrication, characterization and manipulation of nano-range (<100 nm) molecules. The application of nanotechnology in polymers involve the design, manufacturing, processing and application of polymer materials filled with nano-particles and/or devices of nano range (Paul and Robeson, 2008; Danie et al., 2013; Momin and Joshi, 2015). The enormous potential of this promising intervention has gained attention of researchers from multi-disciplinary areas i.e., biological sciences, chemistry, engineering and physics. Owing to high global interest, nanotechnology has been proposed to impact the global economy by around $3 trillion by 2020, generating a requirement of approximately 6 million professionals in different inter-related sectors (Duncan, 2011). As predicted by the Institute for Health and Consumer Protection (IHPC), the nanoparticle based market will touch $20 billion mark by 2020 (Belli, 2012; Montazer and Harifi, 2017). It can be anticipated that nanotechnology will create a major thrust for the development of advanced packaging systems for the sake of consumers. Differently from the materials at macroscale, nanomaterials display specific and improved physicochemical properties. By virtue of their small size, nanoparticles hold a huge surface-to-volume ratio and surface activity. When affixed to desirable polymers, nanomaterials results in improved mechanical strength, electrical conductivity and thermal stability etc. Nanomaterials thus improve the mechanical and barrier properties of food packages; along with offering active and intelligent packaging systems (Mihindukulasuriya and Lim, 2014).

In order to meet effective food packaging requirements, advanced nanomaterial augmented polymers will help to amplify the benefits associated with existing polymers, with enhanced safety, besides addressing environmental concerns. The developed packaging material will contribute in reducing any serious interaction between packaging and food matrices, impact over consumer's health, drop in quantity of waste material, improved biodegradability and barrier shielding to gases and light, and reduced CO_2_ emissions. The ongoing advancement(s) in the area of polymer nanomaterials for food packaging (PNFP) faces a challenge ahead to overcome the safety and regulatory barriers. Upon qualifying the safety parameters and production at a competitive ratio between cost and performance, the new PNFP will be an ideal candidate for widespread application in food packaging (Arora and Padua, 2010; Silvestre et al., 2011; Souza and Fernando, 2016).

**Figure 1:**
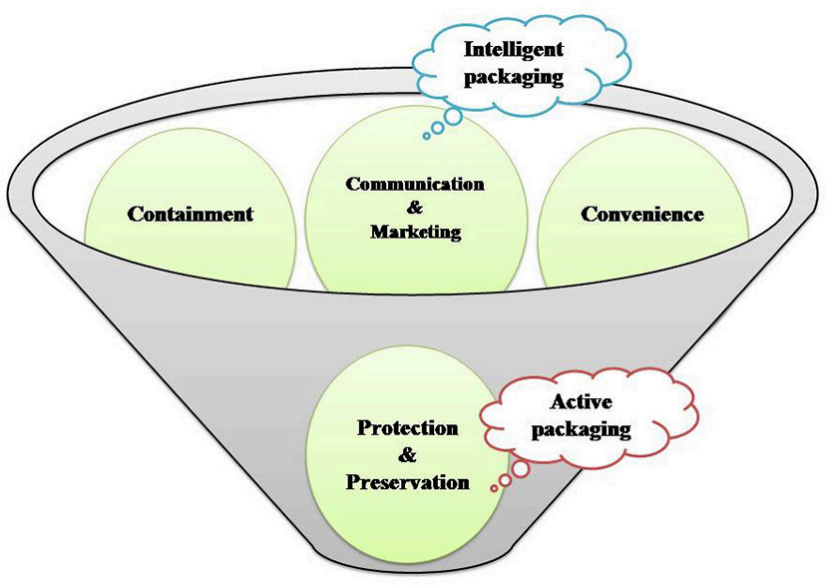
Concept behind and mode of action of active packaging and intelligent packaging.

The introduction of polymer nanotechnology in food packaging aims to improve the principal features of traditional packaging systems i.e., ***containment*** (ease of transportation and handling), ***convenience*** (being consumer friendly), ***protection and preservation*** (avoids leakage or break-up and protects against microbial contaminants, offering longer shelf life), ***marketing and communication*** (real-time information about the quality of enclosed food stuffs, besides the nutritional constituents and preparatory guidelines (Figure 1; Silvestre et al., 2011; Vanderroost et al., 2014).

**Figure 2:**
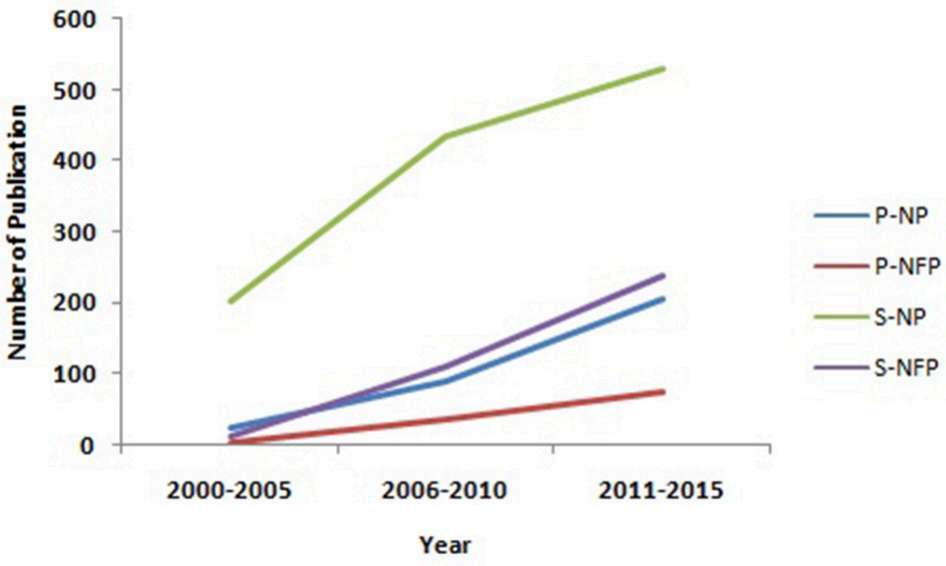
The global trend of research interest in nano packaging (NP) and Nano food packaging (NFP), as estimated by the number of Pubmed (P) and Scopus (S) publications hits from 2000 to 2015 (Source: http://www.ncbi.nlm.nih.gov/pubmed; https://www-scopus-com.scopeesprx.elsevier.com/).

In the twenty-first century, food producers/processors/vendors and consumers, seeks novel and resourceful food packaging systems, with committed food safety, quality and traceability. This demand needs innovative tools/technology that can be assembled together for application in food packaging. The current interventions and developments in food packaging to be commercially feasible and effectively acceptable, must meet regulatory guidelines along with a justified outcome that outweighs the associated expenses of added novel technology (Vanderroost et al., 2014). Figure 2 reflects the growing interest in this field as can be substantiated with the growing number of publications since 2000.

The major drawback associated with food packaging material(s) is their permeable nature. None of the packaging material offers complete resistance to the atmospheric gases, water-vapors and food and packaging material presently. Among the available options organic polymer materials *viz*. polypropylene, polyethylene, polyethylene terephthalate, polystyrene and polyvinyl chloride, remain the main choice material in food packaging industry, favored due to their lesser cost, simple processing and light weight. However, the key problem rests in their inherent permeability to gases and other small molecules.

**Figure 3:**
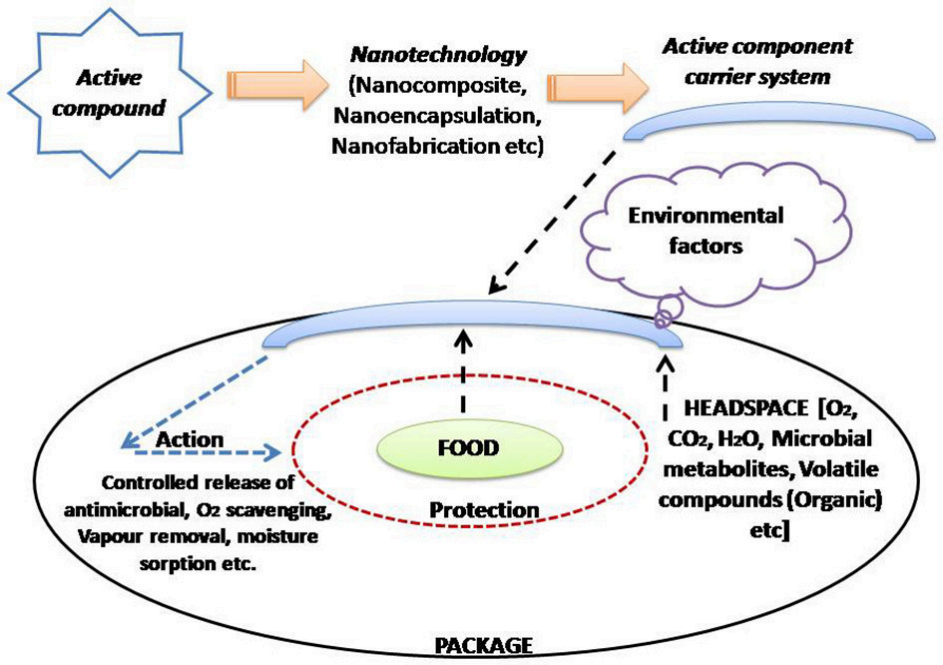
Active packaging and its association with nanotechnology (Adapted from Mihindukulasuriya and Lim, 2014).

**Figure 4:**
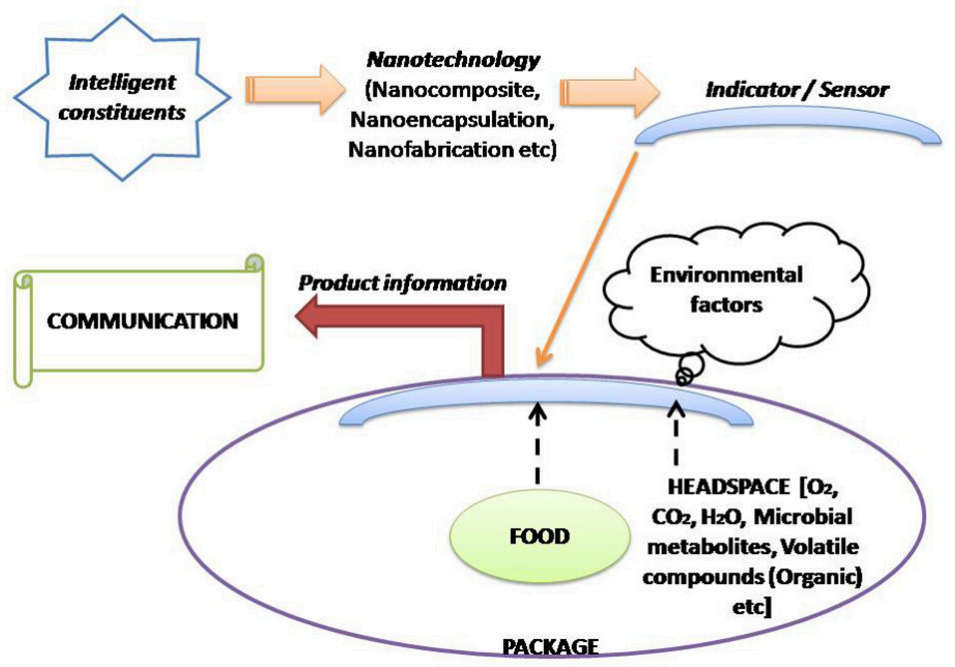
Demonstration of intelligent packaging concept and its association with nanotechnology (Adapted from Mihindukulasuriya and Lim, 2014).

A sole polymer fails to offer all the desirable properties needed for efficient food packaging; so blending of polymer(s) or complex multilayer films is generally preferred (Robertson, 2012). This lacuna can be sorted out by using polymer nano-composites as food packaging materials. Nanomaterials are commonly classified into nanoparticles (NPs), nanoclays (NCs) and nanoemulsions (NEs) which have numerous applications in food sector (Ranjan et al., 2014). Basically, the applications of polymer nanomaterials for food packaging will be discussed under three sections i.e., **improved, intelligent/smart and active packaging**. In case of improved packaging; the nanoparticles application in the polymer matrix improves the elasticity, gas barrier characteristics and stability of temperature/moisture parameters. Intelligent packaging offers superior functionality in terms of communication and marketing. It provides dynamic feedback on the actual quality of packaged food and also acts as a guard against fraudulent imitation. Active packaging assures protection and preservation based on mechanism(s) activated by intrinsic and/or extrinsic factors (Lim, 2011; Silvestre et al., 2011). Active packaging incorporates the active constituent using nanotechnology into a food package material and the carrier component can interact with internal and/or external factors, thereby stimulating actions, which increases the shelf-life, food quality and safety of the food product. However, in case of intelligent packaging systems, the nanotechnology based indicator/sensor(s) are incorporated into the food package, where they can interact with internal (food components and headspace) and external environmental factors (Figures 3, 4; Mihindukulasuriya and Lim, 2014).

## Improved packaging through nanocomposites

Nanocomposites, a fusion of traditional food packaging material with nanoparticles are gaining active interest in food packaging sector. In addition to its remarkable antimicrobial spectrum, it displays great mechanical performance and tough resistant characteristics (Montazer and Harifi, 2017). Nanocomposites are usually made up of a polymer matrix in a continuous or discontinuous phase (Arora and Padua, 2010). It is a multiphase material resulting from the amalgamation of matrix (continuous phase) and a nano-dimensional material (discontinuous phase). Based on the nano-material, the nano-dimensional phase is generally characterized into **nanospheres or nanoparticles**, **nanowhiskers or nanorods, nanotubes and nanosheets, or nanoplatelets** (Bratovcic et al., 2015). Nano-sized phases augment the mechanical properties of polymer, where the elastic strain is transferred to nano-reinforced material. Owing to this property, nanocomposite has been recognized as a gold standard for improvising the mechanical and barrier characteristics of polymers (Othman, 2014). Besides improving the mechanical and barrier characteristics, nanoparticles also append active or smart properties to the packaging system (Duncan, 2011). The application of nanotechnology in polymer science can open new avenues for improving the characteristic features and cost-price-competence of packaging materials (de Azeredo et al., 2011; Malathi et al., 2014).

Polymer nanocomposites (PNCs), the mixtures of polymers with inorganic or organic fillers with particular geometries (fibers, flakes, spheres, particulates) have been recently introduced as novel packaging materials (Prateek et al., 2016). The aspect ratio (the ratio of largest to smallest dimension of filler) of packaging filler material plays a significant role. Fillers having higher aspect ratios possess more specific surface area, with associated high reinforcing properties (Dalmas et al., 2007; Rafieian and Simonsen, 2014). Various nanomaterials such as silica (Bracho et al., 2012), clay (Schuetz et al., 2011), organo-clay (Ham et al., 2013), graphene (Lee et al., 2013), polysaccharide nanocrystals (Lin et al., 2012), carbon nanotubes (Swain et al., 2013), chitosan (Chang et al., 2010), cellulose-based (Sandquist, 2013), and other metal nanoparticles, such as, ZnO_2_ (Esthappan et al., 2013), colloidal Cu (Cardenas et al., 2009), or Ti (Li R. et al., 2011) are being extensively explored as fillers.

## Clay and silicate nanoplatelets

**Figure 5:**
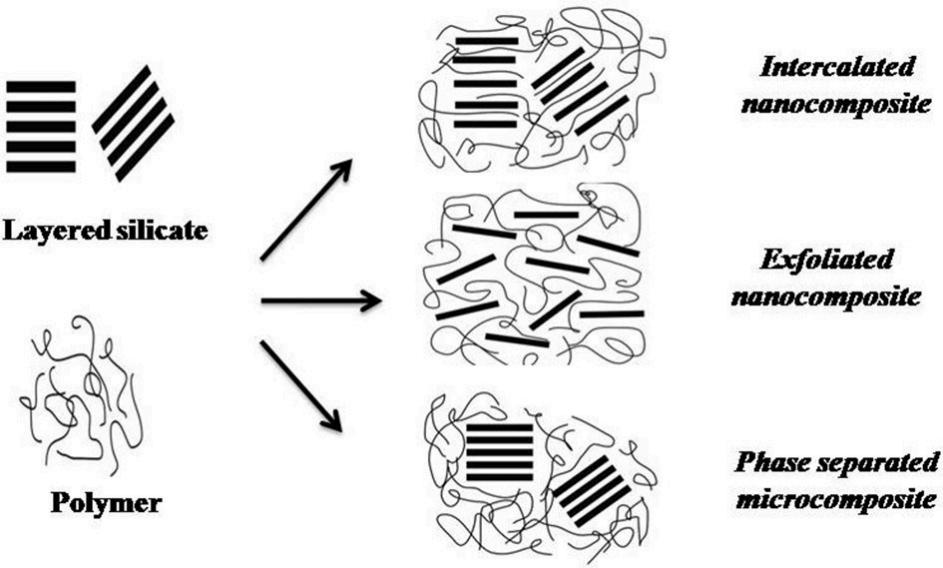
Types of composites obtained from the interaction between layered silicate and polymers (Adapted from de Azeredo, 2009).

Clay and silicates, owing to their availability, low cost and relatively simple processibility have attracted focus of researchers as potential nanoparticles. The layered silicates, about 1 nm thick to a few microns long are usually employed in nanocomposites in their two-dimensional structure. The combination of silicates and polymers impart excellent barrier properties. This combination enhances the diffusive path for an infiltrate molecule (Mirzadeh and Kokabi, 2007; de Azeredo, 2009). In addition to the typical tactoid structure of microcomposites, interaction among layered silicates and polymers may result in intercalated or exfoliated nanocomposites (Figure 5). The intercalated nanocomposites represent a multilayerd structure with alternating polymer/inorganic layers lying apart by few nanometers. Such structures results through the penetration of polymers chains into the interlayer region of clay lead (Weiss et al., 2006; Pradhan et al., 2015). The exfoliated nanocomposites comprises of extensive polymer penetration with random dispersion of clay layers (Luduena et al., 2007). Application of montmorillonite clay as a nanocomponent in extensive variety of polymers such as, polyethylene, nylon, polyvinyl chloride, and starch dates back to 1990s (Montazer and Harifi, 2017). **Montmorillonite (MMT)** [Mx(Al_4_-xMgx) Si_8_O_20_(OH)_4_] is the most common clay filler. It represents an octahedral sheet of Al (OH)_3_ between silica tetrahedral bi-layers (Weiss et al., 2006; Mittal, 2009), linked together by weak electrostatic forces (Tan et al., 2008). The imbalance between the surface negative charges is compensated by the presence of exchangeable cations, Na^+^ and Ca^2+^ (Tan et al., 2008). The clay layers offers resistance to gases and water vapor permeability (Koh et al., 2008; Lotti et al., 2008; Adame and Beall, 2009). Further, addition of clays improves the mechanical strength of biopolymers (Ghanbarzadeh et al., 2015; Gutierrez et al., 2017). The application of 5% (w/w) of clays in thermoplastic starch (TPS)/clay nanocomposites, improves the mechanical properties with decreased water vapor permeability of starch biopolymer (Muller et al., 2012). Clay nanoparticles are further known to impact the glass transition (Petersson and Oksman, 2006) and thermal degradation temperatures (Cyras et al., 2008). Nano-clays also have prospects in active and intelligent food nano-packaging. Recently, Gutierrez et al. (2017) proposed a nano-packaging system developed by inserting blueberry extract between the silicate interlayer spaces of clay. Blueberries possess anthocyanins which changes color with pH, attributed to a shift between quinoidal and flavylium forms. Incorporation of blueberry extract could modify these clays into active and intelligent nanocomposites.

## Cellulose-based nanofibers or nanowhiskers

Cellulose, the building material of long fibrous cells, is a highly strong natural polymer. It is ubiquitous, cost-effective, environmentally friendly and easy to recycle. Cellulose is also explored as a supporting material for many nanomaterials. Application of cellulose increases the surface area of nanoparticles associated with their enhanced activity. These additive features make cellulose nanofibers an attractive class of nanomaterials (Podsiadlo et al., 2005; de Azeredo et al., 2011; Arora et al., 2016). Basically two types of nano reinforcements *viz*. microfibrils and whiskers, can be derived from cellulose. The cellulose chains appear as microfibrils (or nanofibers), which are bundles of molecules that are elongated and stabilized through hydrogen bonding (Wang and Sain, 2007; Kumar and Kumbhat, 2016). Each microfibril is further formed by aggregation of elementary fibrils, which contain crystalline and amorphous parts. The crystalline parts can be isolated by acid hydrolysis treatments; and are referred to as whiskers, nanocrystals, nanorods or rod like cellulose microcrystals (Lima and Borsali, 2004; Chirayil et al., 2014). The dimensions of whiskers, after hydrolysis mainly rely on the percentage of amorphous regions in the bulk fibrils, which differ between different organisms (Gardner et al., 2008). Microcrystalline cellulose (MCC) consists of huge quantity of cellulose microcrystals associated with amorphous areas (Petersson and Oksman, 2006).

Cellulose based nano reinforcements improve the strength, thermal characteristics and modulus of polymers, with restricted elongation (Petersson and Oksman, 2006; Kumar and Kumbhat, 2016). The assimilation of cellulose nanowhiskers and starch systems improve their thermo-mechanical characteristics, along with reduced water sensitivity and intact biodegradability (Lima and Borsali, 2004). Cellulose nano reinforcements also improvised the moisture barrier properties of polymer films (Svagan et al., 2009). Earlier, Ghaderi et al. (2014) prepared an all-cellulose nanocomposite (ACNC) film consisting of sugarcane bagasse nanofiber and N,N-dimethylacetamide/lithium chloride as solvent. The study utilized a very low-value agricultural waste product for preparing a high performance nanocomposite with a tensile strength of around 140 MPa.

## Carbon nanotubes

Carbon nanotubes (CNTs) are available as either single wall nanotube (SWNT) or multiwalled nanotubes (MWNT). SWNT is generally one atom thick, whereas, MWNT comprises of several concentric tubes with very high aspect ratios and elastic modulus (Zhou et al., 2004; Moghadam et al., 2015). The tensile strength/modulus of several polymers such as polyethylene naphtalate, polyvinyl alcohol, polyamide and polypropylene have been improvised by incorporating carbon nanotubes and polyamides (Zeng et al., 2006; Kim J. Y. et al., 2008; Prashantha et al., 2009). CNTs possess elastic modulus of upto1TPa and tensile strength of 200GPa (Lau and Hui, 2002). CNTs exhibit antibacterial properties, attributed to their direct penetration through microbial cells (Kang et al., 2007; Kuswandi, 2017). Dias et al. (2013) documented that CNTs in combination with allyl isothiocyanate could inhibit *Salmonella choleraesuis* for over a period of 40 days of storage. Asgari et al. (2014) reported that CNTs infused with polyethylene films used for the packaging of Mazafati dates, prevented fungal invasion for up to 90 days. Single-walled carbon nanotubes with cobalt meso-arylporphyrin complexes has also been explored for developing a chemiresistive detector, which detects amines generated during spoilage of meat (Liu et al., 2015). However, in spite of having wide application, concerns associated with their processing and dispersion aspects, along with high cost, limits their incorporation in nanocomposites (Arora and Padua, 2010; Moghadam et al., 2015).

## Starch nanocrystals

Starch has been extensively explored over decades as a choice material for food packaging applications. Several associated benefits *viz*. abundance, biocompatibility, non-toxicity, low cost, biodegradability, easy availability, and stable in air (Dandekar et al., 2012; Wesley et al., 2014) further enhances its possible applications. The incorporation of inorganic materials and synthetic polymers further improves its water resistance properties (Cyras et al., 2008). Native starch granules can be submitted to an extended-time hydrolysis at temperatures below the gelatinization temperature when the amorphous regions are hydrolyzed allowing separation of crystalline lamellae, which are more resistant to hydrolysis. The starch crystalline particles show platelet morphology with thicknesses of 6–8 nm, improve tensile strength and modulus of pullulan films, with decreased elongation property (Kristo and Biliaderis, 2007; Arora and Padua, 2010). It has been proposed that the positive charge present over any antimicrobial agent contributes to its antimicrobial activity. As a result, the antimicrobial spectrum of metals incorporated/adsorbed on to polysaccharides surface increases, owing to enhanced surface area (Arora et al., 2016).

## Chitin/chitosan nanoparticles

Chitosan, a heteropolysaccharide is known for its biocompatibility, biodegradability, along with metal complexation. The polycationic nature of chitosan is mainly accountable for its wide antimicrobial activity (Arora et al., 2016). Chitosan nanoparticles are formed through ionic gelation, where the positively charged amino groups of chitosan electrostatically interacts with the polyanions engaged as cross-linkers (Lopez-Leon et al., 2005; Ahmed and Aljaeid, 2016; Kumar and Kumbhat, 2016). Earlier, Lu et al. (2004) observed that the combination of chitin whiskers with soyprotein isolate (SPI) thermoplastics significantly enhanced the tensile characteristics of the matrix and their resistance to water. Later, Burdock (2007) proposed hydroxypropyl methylcellulose (HPMC) act as a potential material for edible packaging films. De Moura et al. (2008) added that the chitosan based nanocomposites in HPMC helped in the improvement of the mechanical and barrier characteristics. Tripathi et al. (2009) developed a chitosan-based antimicrobial film comprising of chitosan and polyvinyl alcohol. The developed film could effectively extend the shelf-life of tomato, displaying antibacterial activity against *E. coli, S. aureus*, and *B. subtilis*. Edible films and coatings prepared from chitosan improvised physicochemical and microbiological quality of fresh-cut vegetables and fruits (Ce et al., 2012; Ramos et al., 2012). The active antibacterial bags consisting of chitosan/polyethylene have displayed efficacy in inhibiting total aerobic mesophilic bacteria, coliforms, molds, and yeasts in chicken drumsticks; besides maintaining pH, color, and hardness of samples (Soysal et al., 2015). Chitosan/silver, chitosan/gold and chitosan/cinnamaldehyde nanocomposite films have demonstrated antimicrobial activity against *E. coli, S. aureus, P. aeruginosa, Aspergillus niger*, and *Candida albicans* (Youssef et al., 2014; Rieger et al., 2015).

## Active packaging

Unlike conventional food packaging, an active packaging is intentionally designed packaging system that incorporates components that would release (antimicrobial or antioxidant agents) or absorb (oxygen or water vapor) material into or from the packaged food or the food environment. Amalgamation of active compounds *viz*. antimicrobial agents, preservatives, O_2_ and water vapor absorbers, ethylene removers etc. with polymer renders it more effective for boosting the shelf life and quality of food product (Arora and Padua, 2010; Ranjan et al., 2014; Majid et al., 2016).

## Inorganic nanoparticles

**Figure 6:**
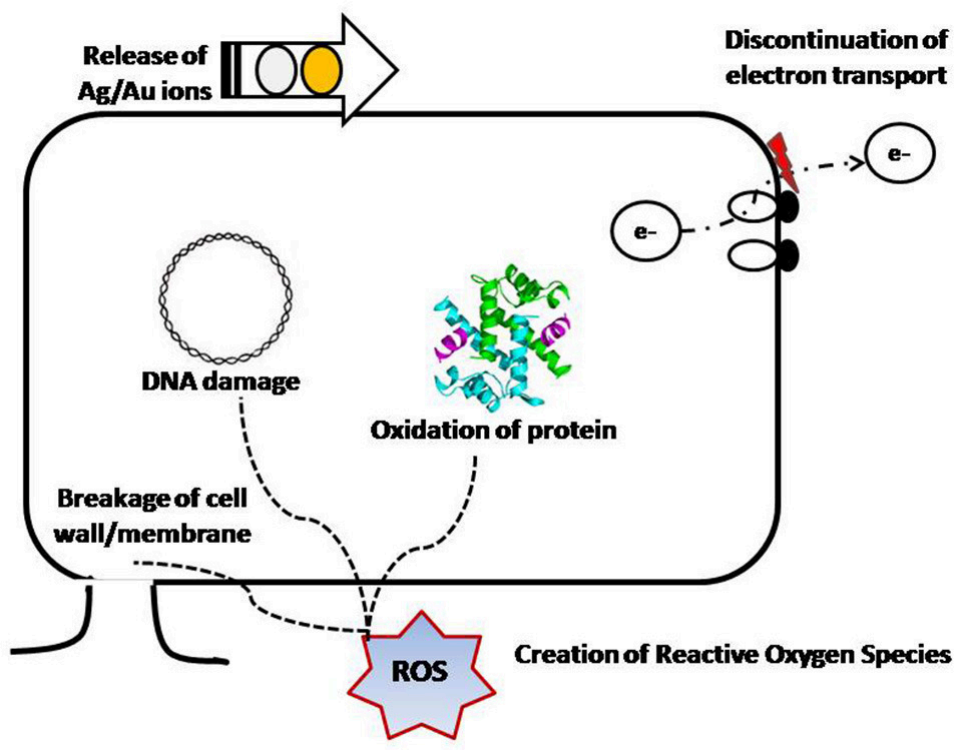
Different mechanisms of antimicrobial activity exhibited by nanoparticles (Modified from Li et al., 2008).

Various metals [Silver (Ag), Gold (Au), Zinc (Zn)] and metal oxide [Titanium dioxide (TiO_2_), zinc oxide (ZnO), silicon oxide (SiO_2_) and magnesium oxide (MgO)] derived nanomaterials have been explored in diverse active packaging applications (Bikiaris and Triantafyllidis, 2013). These particles either function on direct contact or they can migrate slowly and react preferentially with organics present in the food. Nanoparticles antimicrobial activity might be due to one of these mechanisms: direct interaction with the microbial cells (interrupting trans-membrane electron transfer, disrupting/penetrating the cell envelope); oxidizing cell components; and production of secondary products (e.g., reactive oxygen species - ROS or dissolved heavy metal ions), leading to cell damage (Figure 6) (Li et al., 2008).

### Silver nanoparticle

Silver nanoparticles are among the most explored nanoparticles, owing to their established antimicrobial potential against multiple commensal and pathogenic strains (Kumar and Munstedt, 2005). Besides bacterial strains, silver nanoparticles are known to be inhibitory against multiple fungi and also several viruses (HIV and monkeypox; Duncan, 2011). Silver targets bacterial metabolism by binding to its DNA, proteins and enzymes; resulting into bacteriostatic effects (Cavaliere et al., 2015). Silver nanoparticles destabilize and disrupt both the outer and cytoplasmic membranes (Morones et al., 2005). Silver nanoparticles also inhibit the respiratory chain enzymes and can also stimulate the production of reactive oxygen species (ROS) (Emamifar et al., 2011b). Earlier, Damm et al. (2008) evaluated the effectiveness of polyamide 6 and silver micro and nanoparticles incorporated films against *E. coli*. The developed nano-particles based film completely inhibited the *E. coli* growth. However films with silver micro particles killed 80% of the bacteria. Rhim et al. (2014) reported noteworthy antimicrobial activity of silver nanoparticles incorporated agar films against *Listeria monocytogenes* and *E. coli* O157:H7. In an earlier study, Busolo et al. (2010) documented strong antibacterial activity of PLA/silver-OMMT nanocomposite against *Salmonella* spp. Antibacterial activity of nanocomposite systems such as PVA dispersed with cellulose nanocrystals and silver nanoparticles has been displayed against *E. coli* and *Staphylococcus aureus* (Fortunati et al., 2013a,b; Sadeghnejad et al., 2014). Lin and co workers analyzed the antimicrobial effect of cellulose/chitosan-silver and cellulose/chitosan nanocomposite films and reported better activity in films with silver nanoparticles (Lin et al., 2015). Fayaz et al. (2009) studied biosynthesized silver nanoparticles incorporated into sodium alginate films in food packaging, and showed noteworthy antibacterial effect against *E. coli* and *S. aureus*. In another study conducted by Sanchez-Valdes et al. (2009), silver nanoparticles deposited over multilayered linear low density polyethylene (LLDPE) showed 70% reduction of *Aspergillus niger*. Recently, Hasim et al. (2015) observed the UV/ozone treated commercial low-density polyethylene (LDPE) films coated by layer-by-layer (LbL) method by alternating the deposition of polyethyleneimine (PEI) and poly(acrylic acid) (PAA) polymer solutions and antimicrobial silver. They demonstrated that the resultant films containing antimicrobial Ag NPs could be explored in antimicrobial packaging. De Moura et al. (2012) reported that size of nanoparticles also affects the antimicrobial efficacy as they evaluated the antibacterial properties of hydroxypropyl methylcellulose films containing silver nanoparticles with diameters of 41 and 100 nm against *E. coli* and *S. aureus* and observed that silver nanoparticles with smaller size (i.e., 41 nm) had greater antibacterial properties than the larger one.

Silver zeolites are also used to create antibacterial polymer composites. AgNP-based nanocomposites are stable and offer slow release of silver ion into stored foods, resulting in persistent antimicrobial activity. In a study where AgNP/SiO_2_ nanocomposite material was compared with that of Ag zeolite and AgNO_3_/SiO_2_ composite, effective antimicrobial activity was displayed by both the materials; however, longer period of activity was displayed by the nancomposite; while a better immediate effect was observed with zeolite-based material. Nanocomposite based silver nanoparticles may find application in food packages requiring longer transportation or storage. To explore the antimicrobial potential of AgNP on the shelf life of food, various AgNP/polymer nanocomposite materials have been inspected within actual food systems. Fayaz et al. (2009) proposed an edible antibacterial film of alginate and AgNPs over sterilized carrots and pears. An et al. (2008) documented that coating of fresh asparagus spears with AgNP/polyvinyl pyrrolidone nanocomposite films, extended its shelf life by 25 days at refrigerated storage. When the Chinese jujube fruit was stored in bags made up of AgNP/nanoparticulate TiO2/polyethylene films, lesser decaying rate along with delayed ripening over a period of 12 days was observed (Li et al., 2009). Orange juice stored at 4°C in LDPE films having P105 (TiO_2_ and 10 nm nanosilver mixture) displayed considerable reduction in *Lactobacillus plantarum* growth over the storage period of 112 days (Emamifar et al., 2011a). Recently, Orsuwan et al. (2016) prepared binary blend films comprising of agar and banana powder (A/B) composite films reinforced with silver nanoparticles (A/B/AgNPs). These films displayed strong antibacterial activity against *E. coli* and *L. monocytogenes*. With the increase in the concentration of banana powder, characteristics such as UV light absorption, water vapor barrier and antioxidant activity of A/B blend also amplified whereas their mechanical properties decreased. Fernandez et al. (2010a) observed another kind of application i.e., AgNPs containing cellulose pads, which reduces the levels of microbial exudates of meat stored in modified atmosphere packaging. In a follow up study, antimicrobial activities of AgNP-containing cellulose pads were displayed in fresh foods, along with slower ripening rates with extended shelf lives (Fernandez et al., 2010b). Further, cellulose pads containing silver nanoparticles have also been successfully applied for beef coating, wherein significant reduction in microbial load was reported (Smolkova et al., 2015).

### Other antimicrobial nanoparticles

Copper nanoparticles were shown to inhibit the growth of *Saccharomyces cerevisiae, E. coli, S. aureus* and *L. monocytogenes* on a polymer composite after 4 h exposure (Cioffi et al., 2005). Sheikh et al. (2011) also revealed good antibacterial effect of copper nanoparticles against *E.coli* and *B. subtilis* in polyurethane nanofibers containing copper nanoparticles. Copper nanoparticles leads to multiple toxic effects such as, generation of reactive oxygen species (ROS), lipid peroxidation, protein oxidation and DNA degradation; which might be responsible for its antimicrobial activity (Chatterjee et al., 2014). Zinc nanocrystals have also been used as an antimicrobial and antifungal agent, when incorporated with plastic matrix (Vermeiren et al., 2002). Different nanoparticles oxide(s) such as **titanium dioxide (TiO**_2_**), zinc oxide (ZnO), silicon oxide (SiO**_2_**)**, and **magnesium oxide (MgO)** do found application in food packaging, due to their ability to act as UV blockers and photo-catalytic disinfecting agents (Fujishima et al., 2000). Among all, TiO_2_ particles have been most promising (Kong et al., 2010; Farhoodi, 2016). The antimicrobial activity of TiO_2_ nanoparticles is photocatalyzed and these antimicrobial particles are active only in the presence of UV light. TiO_2_ nanoparticles displayed good activity against *S. choleraesuis, Vibrio parahaemolyticus*, and *L. monocytogenes* under UV illumination but not in the dark (Robertson et al., 2005).

### Titanium dioxide (TiO_2_)

Naturally, titanium dioxide exists in three primary phases i.e., anatase, rutile, and brookite; having varied crystal sizes (Naicker et al., 2005). TiO_2_ possess photocatalytic abilities and at nanoscale TiO_2_ shows surface reactivity, which connects it with biological molecules (phosphorylated proteins and peptides) and DNA (Liang et al., 2006; Brown et al., 2008). The surface energy of TiO_2_ nanoparticles amplifies with size and is the significant factor in polymer/filler interaction (Naicker et al., 2005). The surface energies of rutile particles are higher than those of anatase particles of similar size. TiO_2_ is being highly explored in preparing several nanomaterials *viz*. nanoparticles, nanorods, nanowires, nanotubes, mesoporous and nanoporous TiO_2_ containing materials (Chen and Mao, 2006). The antibacterial properties of TiO_2_ is well known (Macwan et al., 2011; Montazer and Seifollahzadeh, 2011) however, the antibacterial capacity of nano-TiO_2_ particles confined to the exposure of UV irradiation (Shi et al., 2008). Although, the exact mechanism of biocidal activity of TiO_2_ is unclear, it may be attributed to its initial oxidative attack over the outer/inner bacterial cell membrane, alterations of Coenzyme A-dependent enzyme activity, and DNA damage through hydroxyl radicals (Kubacka et al., 2014).

Cerrada et al. (2008) examined the photo-activated biocidal properties of TiO_2_ nanoparticles based EVOH films against nine microorganisms (*B. stearothermophilus, S. aureus, E. coli, P. fluorescens, Bacillus* sp., *L. plantarum, E. caratovora, P. jadinii, Z. rouxii*). The TiO_2_ nano-particles were uniformly dispersed ultrasonically. They reported over 5 log reduction for *B. stearothermophilus, Bacillus* sp., *L. plantarum* and *P. jadinii* after 30 min of irradiation in the presence of the TiO_2_/EVOH materials. Chawengkijwanich and Hayata (2008) observed 3 log reduction in *E. coli* count over fresh cut lettuce treated with TiO_2_ nanoparticles coated oriented-polypropylene (OPP) film after 3 h of illumination. In contrary, uncoated films could reduce the *E. coli* count by only 1 log under similar conditions. In another study, TiO_2_ nanoparticle coated plastic films were evaluated against *Penicillium expansum* spoilage in apple, tomato and lemons. Results indicated that the growth of *P. expansum* was suppressed due to the photocatalytic properties of TiO_2_ particles upon exposure to light (Maneerat and Hayata, 2006). Combination of TiO_2_ nanoparticles with silver has been shown to enhance the antimicrobial properties (Li et al., 2009; Wu et al., 2010) to a significant level.

### Silicon oxide (SiO_2_)

Silica nanoparticles (nSiO_2_) also have potential to improve the mechanical and/or barrier properties of various polymer matrices. Wu et al. (2002) examined the tensile characteristics (i.e., strength, modulus and elongation) of nSiO_2_ incorporated polypropylene (PP) matrix. Addition of nSiO_2_ into starch matrix could improve the tensile properties along with decreased water absorption by starch (Xiong et al., 2008). Vladimiriov et al. (2006) integrated nSiO_2_ in an isotactic polypropylene (iPP) matrix using maleic anhydride grafted polypropylene (PP-g-MA) and this nSiO_2_ enhanced the storage modulus of iPP and making the material stiffer with improvised O_2_ barrier capacity of matrix. Jia et al. (2007) generated polyvinyl alcohol and SiO_2_ nanocomposites through radical copolymerization of vinyl silica nanoparticles and vinyl acetate. The resultant nanocomposites exhibited enhanced thermal and mechanical properties, in comparison to pure polyvinyl alcohol. This feature may be due to strong covalent interactions between nSiO_2_ and the polymer matrix. In another study, Tang and Liu (2008) fabricated starch/PVOH/nSiO_2_ biodegradable films and documented that the tensile and water resistance properties of films enhanced with an increase in their nSiO_2_ content. Additionally, increased intermolecular H bonding and formation of C–O–Si groups between nSiO_2_/starch and nSiO_2_/PVOH were observed. Such bonding resulted in improvised miscibility and compatibility between the film components.

Food packaging applications of nano-silica has been explored as food contact surface materials (Chaudhry and Castle, 2011). Bayer Polymers, Germany made silicate nanoparticles enriched packaging film which retards the entrance of O_2_ and other gases and loss of moisture; preventing food spoilage. Nanocor Inc, Chicago, IL, USA, developed a clay nanoparticle based nanocomposite (Advantage Magazine, 2004). Salami-Kalajahi et al. (2012) reported that nanocomposites comprising of 5% silica nanoparticles lead to the improvement of mechanical and physical properties. Recently, Farhoodi (2016) showed that the application of SiO_2_ nanoparticles as fillers in food packaging materials leaves a twisting pathway for gases. Chen et al. (2013) modified paper to form a lotus like super hydrophobic surface by coating with R812S silica nanoparticles and Polydimethylsiloxane (PDMS) silicone oil. The coated paper displayed strong water repellent characteristic.

### Zinc oxide (ZnO)

Zinc oxide particles display good antibacterial activity, which further enhances with decrease in particle size. ZnO requires visible light for stimulation (Yamamoto, 2001; Jones et al., 2008). Its direct contact with microbial cell wall may result in destruction of bacterial cell integrity, liberation of antimicrobial ions i.e., Zn^2+^ ions, and generation of ROS (Sirelkhatim et al., 2015). Sawai (2003) observed that ZnO was the most effective antibacterial agent for *S. aureus* growth inhibition when compared to MgO and CaO. Sevinc and Hanley (2010) explored that ZnO lessen the growth of bacterial biofilms (*Streptococcus sobrinus*) nearly 80% in dental materials. In an another study, Jin et al. (2009) observed different approaches i.e. powder, film, PVP capped and coating; for the application of nano-ZnO in food systems and concluded that nano-ZnO displayed antibacterial effects against *L. monocytogenes* and *Salmonella enteritidis* in liquid egg white and in culture media. Li et al. (2009) compared antimicrobial activity of ZnO powder and ZnO nanoparticles against food borne pathogens (*Bacillus cereus, E. coli, S. aureus, S. enteridis*) and observed that ZnO nanoparticles displayed better antibacterial activity than non-nano powder against all the tested bacteria. Petchwattana and Naknaen (2015) prepared PBS (polybutylene succinate)/thymol film and evaluated its antimicrobial activity against *E. coli* and *S. aureus*. Recently, Petchwattana et al. (2016) displayed significant antibacterial activity of poly butylenes succinate (PBS)/zinc oxide composite films against *E. coli* and *S. aureus*. Wiburanawong et al. (2014) analyzed the inhibitory activity of carvacrol oil against *S. aureus* and *E. coli*. Essential oils act can as a promising antimicrobial agent in PBS film but their unpleasant odors affected its acceptance. To overcome its odor, when ZnO is added to PBS, then the resultant product displayed better antibacterial activity devoid of any odor from volatiles (Li and Li, 2010; Murariu et al., 2011; Zaman et al., 2012).

### Oxygen scavengers

Access to O_2_ in the food package reduces the shelf-life of product owing to diverse degradation processes such as browning, rancidity, growth of aerobic microflora, lessening of vitamins, off-odors, off-flavors and undesirable color changes. Along with it, during storage, fresh fruits and vegetables produces some undesirable compounds i.e., production of ethylene vapor in climacteric fruits, which accelerates the postharvest ripening and shortening the shelf-life. In turn to circumvent these detrimental effects, the entry of O_2_ should be eradicated within the package or lessened to acceptable level according to the product. The content of O_2_ in the headspace of packaged food can be reduced through vacuum sealing or by generating inert gas atmosphere in the packaging (N_2_, CO_2_) or both, and through the use of oxygen absorbent materials (Prasad and Kochhar, 2014). Various active packaging perceptions based on nanocomposite films have been explored for oxygen and ethylene scavenging or limit the path of oxygen diffusion (Mihindukulasuriya and Lim, 2014; Echegoyen, 2015; Montazer and Harifi, 2017). A varied number of polymers having nanostructured oxygen scavengers can be explored as active packaging material for sliced meat, poultry, beverages, cooked pasta, ready-to-eat snacks, fish etc. (Neethirajan and Jayas, 2011). Xiao et al. (2004) developed O_2_ scavenger films and incorporated TiO_2_ NPs to different polymers and this nanocomposite material was further explored as packaging films in O_2_ sensitive food stuffs. Later on, Busolo and Lagaron (2012) modified the high density polyethylene (HDPE) films by integrating them with iron containing kaolinite to produce O_2_ scavenging packaging films. At nanoscale, TiO_2_ can be photoinduced by UV radiation and electrons got accumulated on the TiO_2_ surface. In photocatalytic reactions, the transfer of electron to oxygen in the air is the rate-determining step and the photogenerated holes (h+) have strong oxidation potential, which can react with water and other compounds. Xiao et al. (2004) revealed that the deposition of nanocrystalline TiO_2_ on glass and acetate films, formed a deoxygenated closed environment and the photocatalytic properties of TiO_2_ utilized for the removal of ethylene vapor to delay the ripening of climacteric fruits. Maneerat and Hayata (2006) developed TiO_2_ coated polypropylene films for the removal of ethylene vapor in packaged horticultural products. Ye et al. (2009) explored the photoelectrocatalytic degradation of ethylene using TiO_2_ supported on activated carbon; the membrane obtained is capable of degrading ethylene vapor when irradiated with UV radiation. This strategy can be explored for delaying the ripening of climacteric fruits by constantly removing the ethylene vapor during long-distance transportation.

### Enzyme immobilization

Enzymes possesses multifunctional role in food industry but possess few limitations i.e., sensitivity to processing atmosphere (temperature, pH) and/or to enzyme inhibitors, sometimes restricts their applicability in food system but the concept of immobilization and incorporation of enzymes in packaging materials provides an alternative to the direct use of enzymes in food matrix (Ranjan et al., 2014). Immobilization at nanoscale increases the surface area and boosts the performance by enhancing the stability to pH, temperature and resistance to proteases; besides helping controlled release of enzymes into food system (Rhim et al., 2013; Brandelli et al., 2017). According to Lopez-Rubio et al. (2006), immobilization is an efficient way to improve the stability of enzyme toward pH and temperature, resistance to proteases and other denaturing components and provides an ample environment for their repetitive use or controlled discharge in food system. Fernandez et al. (2008) observed that the enzymes (lactase or cholesterol reductase) inclusion to packaging substances might enhance the food product values and match the consumer requirement with enzyme deficiencies. Rhim and Ng (2007) explored the approach of enzyme adsorption into nanoclays incorporated to polymers. It is further supported by Gopinath and Sugunan (2007) that nanoclay has a high affinity for protein adsorption and could be utilized as a competent enzyme carrier. Ahuja et al. (2007) reported that conductive polymers may be explored as immobilizing matrices for biomolecules. However, Sharma et al. (2004) also validated the same by immobilizing the glucose oxidase onto films of poly (aniline-co-fluoroaniline). Qhobosheane et al. (2001) modified SiO_2_ nanoparticles to immobilize glutamate dehydrogenase and lactate dehydrogenase, which displayed better enzyme activity after immobilization.

## Intelligent/smart packaging system

Intelligent or smart packaging systems boost the communication aspect of a package and such type of advanced packaging recognize any characteristics of the packaged food and apply different mechanisms to register and convey information regarding the existing quality or condition of the food with regard to its safety and digestibility. Such system uses different innovative communication methods i.e., Nano-sensors, time-temperature indicators, oxygen sensors, freshness indicators etc. (Kerry et al., 2006; Bouwmeester et al., 2007; Majid et al., 2016). The inclusion of nanosensors in food packaging systems helps in detecting the spoilage associated changes, pathogens and chemical contaminants, and thus providing the exact scenario of food products freshness (Liao et al., 2005). Nanosensors are nanotechnology enabled sensors characterized by a range of variations. Generally, nanosensors can be applied as labels or coatings to add an intelligent function to food packaging in terms of ensuring the integrity of the package through the detection of leaks (for foodstuffs packed in a vacuum or inert atmosphere), indications of time–temperature variations (e.g., freeze–thaw–refreeze), or microbial safety (the deterioration of foodstuffs) (Mahalik and Nambiar, 2010; Watson et al., 2011; Fuertes et al., 2016). Gas sensors are basically used for revealing the gaseous analyte in the package. Optical O_2_ sensors works on the principle of luminescence quenching or absorbance changes caused by direct contact with the analyte; whereas optochemical sensors are used to check the quality of products by sensing gas analytes such as, hydrogen sulfide, CO_2_ and volatile amines (Biji et al., 2015). The unique chemical and electro-optical properties of nanoscale particles respond to environmental changes (e.g., temperature or humidity in storage rooms, levels of oxygen exposure), product degradation or microbial contamination (Bouwmeester et al., 2009). Therefore, such technology would apparently benefit consumers, industry stakeholders and food regulators (Duncan, 2011).

## O_2_ sensors

Food packaging systems with restricted oxygen availability are preferred over those giving free oxygen accesses. In order to achieve this packaging needs to be carried out under vacuum or nitrogen gas along with incorporation of irreversible O_2_ sensors. In case of modified atmosphere packaging (MAP), the headspace O_2_ concentration is either deliberately reduced to an optimal level or entirely eliminated as per food product requirements. Lee et al. (2005) developed a UV based colorimetric O_2_ indictor that utilizes TiO_2_ nanoparticles for photosensitization of triethanolamine induced reduction of methylene blue in a polymer encapsulation medium. UV irradiation leads to bleaching of sensor, which remains colorless until exposed to O_2_. The pace of change in color is relative to the level of O_2_ exposure (Gutierrez-Tauste et al., 2007). Mills and Hazafy (2009) used nanocrystalline SnO_2_ as photosensitizer and a colorimetric O_2_ indicator consisting of glycerol (as electron donor), methylene blue (a redox dye) and hydroxyethyl cellulose (as encapsulating polymer). Upon exposure to UV-B light, indicator gets bleached and photoreduction of dye takes place by SnO_2_ nanoparticles. Another study done by Mihindukulasuriya and Lim (2013) generated UV based activated O_2_ indicator membrane through electrospinning method. Encapsulating of the active components *viz*. TiO_2_ nanoparticles, glycerol and methylene blue within electrospun poly fibers, enhanced the oxygen sensitivity of the membrane.

## CO_2_ sensor

To monitor freshness or quality of packaged foods during their storage condition, Jung et al. (2012) formed chitosan-based CO_2_ indicator. For the enhancement of signal strength of indicator, 2-amino-2-methyl-1-propanol (AMP) was used as an additive to the chitosan solution. The indicator solution of chitosan and AMP can be easily packed into sachets, which are permeable to gaseous CO_2_ inside food packages. Borchert et al. (2013) develop an optochemical CO_2_ sensor which uses a phosphorescent reporter dye phosphorescent Pt-porphyrin PtTFPP and a colourimetric pH indicator (naphtholphthalein) integrated in plastic matrix together with a phase transfer agent (tetraoctyl or cetyltrimethyl ammonium hydroxide). The sensor material was optimized for food packaging applications and underwent complete characterization with respect to its CO_2_ sensitivity and cross-sensitivity to O_2_. Puligundla et al. (2012) concluded that the optical CO_2_ gas sensors, particularly, dry optical sensors having pH-sensitive dye indicators can be explored for food packaging applications.

## Detection of spoilage and pathogenic microorganisms

According to CDC (2011), approximately 48 million cases of food-borne illnesses are reported per year in USA. It was estimated that reduction in incidence of food borne infection by even 1% would benefit about 500,000 individuals (Scallan et al., 2011). Food borne illness caused by bacteria, fungi or viruses remains a matter of public health concern. The need of hour is to develop a detection method which is rapid, precise, and cost effective (Duncan, 2011). Currently followed detection methods mainly explore immunological assays dependent over the selective interaction between antibody and antigen. Microbial sensors exploring nanoparticles do also work on similar approach besides having unique optical and electrical characteristics in conjunction with other properties such as spacious and simply functionalized surfaces.

Antibodies conjugated to nanomaterials such as quantum dots (QD) are mainly explored for detection of bacteria. QDs are mainly employed due to their characteristic high fluorescence efficiency, stability for photobleaching, high sensitivity, extended decay life-time and electronic properties such as, wide and continuous absorption spectra and narrow emission spectra (Valizadeh et al., 2012). Yang and Li (2006) explored QDs for detection of *E. coli* O157:H7 and *Salmonella* Typhimurium. Highly fluorescent CdSe/ZnS QDs were conjugated to anti *E. coli* O157 and anti-*Salmonella* antibodies. QDs for *E. coli* and *Salmonella* had different emission wavelength but shared common excitation wavelength, allowing simultaneous detection of the two test pathogens. The QD conjugate method has several benefits over typical fluorescence dyes. QDs possess significantly higher fluorescence intensity, offer resistance to photobleaching and have additional advantage of multiplexed detection using different QDs (Mihindukulasuriya and Lim, 2014).

QDs and organic fluorescent compounds (OFC) are classified into down-conversion phosphors and up-conversion fluorescent nanoparticles (UCNPs). The down-conversion phosphorus absorbs energy at low wavelength and emits radiation at higher wavelength whereas; UCNPs get excited by low energy radiations (near-infrared) and emit higher energy visible radiation. These characteristic features help them to rule out autofluorescence and photodamage related problems. UCNPs are extremely sensitive sensors that can also be used for bacteria, enzyme, protein, nucleic acid, pH, NH_3_, CO_2_, and other analytes (Gnach and Bednarkiewicz, 2012). Ong et al. (2014) conjugated *E. coli* specific antibodies to citrate modified oleic acid-capped NaYF4:Yb, Er UCNPs for selective detection of *E. coli*. Earlier, up-conversion conjugates technique was used to design a quick lateral flow test, for *E. coli* detection (Niedbala et al., 2001). It is also applied for multiplex detection of other drug compounds. QDs and UCNPs have found application in imaging, labeling and medicine. Further, they can have potential application in food packaging; particularly for awaited advancement in intelligent label for pathogen and toxins detection in food matrix. Therefore, more R&D needs to be done to further establish this technique in food packaging application.

## Freshness indicators

Freshness indicators aim to provide the actual information regarding the quality of food product during storage, transit and display. The reaction between microbial metabolites and incorporated indicators gives visual information about the microbial quality of product (Biji et al., 2015). The incorporated freshness indicators are sensitive to spoilage compounds or microbial metabolites generated during spoilage of food product, for example volatile sulfides and amines (Mihindukulasuriya and Lim, 2014; Fuertes et al., 2016). The approach for the recognition of pathogens in food system depends upon the isolation of pathogens from the food environment (Rossi et al., 2017). Smolander et al. (2004) detects the spoilage of meat products by depositing a transition metal (silver or copper) coating (1–10 nm thick) over plastic film or paper packaging structures. This coating turns dark upon reacting with sulfide volatiles produced from fresh meats undergoing spoilage. Maynor et al. (2007) develop a conjugated poly (thiophene) to produce multi-dimensional colorimetric effects that distinguish 22 structurally related amines with high (97%) accuracy. The conjugated system was applied in detection of amine profiles in tuna fish. Later, a polyaniline film sensitive to a group of basic volatile amines released during spoilage of fish was developed. Such interventions are useful in real-time detection of fish spoilage at different temperatures (Kuswandi et al., 2012). Lim et al. (2013) developed a peptide receptor based portable bioelectronic nose for analyzing food freshness by targeting trimethylamine, the key volatiles produced during raw seafood spoilage. Esser et al. (2012) developed a sensor using carbon nanotubes to detect CO_2_, volatile compounds and ethylene emitted during the ripening of fruits.

## Time-temperature indicators (TTIS)

Chiefly, the temperature exploitations meet by food during transit and distribution are one of the main environmental factors responsible for the reduced shelf-life of foods. To counter different temperature abuses, TTIs are useful to monitor the thermal history during food storage, handling, and distribution. TTIs allow the retailers to ensure that the foods have been stored at the suitable temperatures; help consumers in determining the quality of food product they are purchasing; and help manufacturers in monitoring of supplied foodstuff throughout the supply chain (Mihindukulasuriya and Lim, 2014; Ranjan et al., 2014).

Singh (2000) grouped TTIs into three basic types *viz*. abuse indicators, partial temperature history indicators and full temperature history indicators. Abuse indicators designate attainment of a particular temperature. Partial temperature history indicators present the time–temperature history only if the temperature surpasses a critical set limit. In contrary, the full temperature history indicators provide a constant monitoring of temperature changes with time. The communication regarding the change in food quality is generally linked with color development associated with a temperature dependent migration of dye through a porous material; or change in color of an indicator. Timestrip, an AuNP based iStrip for chilled foods appear red at temperatures beyond freezing. However, accidental freezing leads to irreparable agglomeration of the AuNP, resulting in loss of the red color (Robinson and Morrison, 2010). Zeng et al. (2010) developed triangular Ag nanoplates as colorimetric indicators for monitoring the time-temperature history, based on thermodynamic un-steadiness of Ag nanoplates. The key problem regarding the application of TTIs in food is related to the kinetics of TTIs response, it should match with those of the key degradation reactions in food. In TTI systems, its synchronization and degradation kinetics is a matter of concern as their reaction cannot be programmed readily. In concern to above problem, Zhang et al. (2013) formed a TTI working on a reaction of epitaxial overgrowth of Ag shell on Au nanorods dipped in cetyl-trimethylammonium chloride (CTAC) solution appears red in color because of two absorbance bands arise from transverse and plasmon resonances, respectively. With the introduction of AgNO_3_ and a reducing agent (ascorbic acid), Ag atoms get deposited on Ag nanorods and form the shell of Au/Ag nanorods. The extinction bands of longitudinal plasmon resonance transfer to lesser wavelengths as the Ag shell thickens which leads to changes in color from red, yellow and lastly green. Kinetics of this color variation can be managed by altering the Au nanorods, CTAC, reducing agent and pH value. Those products which exhibited diverse degradation kinetics can be managed by such type of low cost programmable indicator.

## Humidity indicators

Monitoring of humidity will provide an edge to know about the integrity of the package and actual status regarding the quality and safety of the food items in the package. To overcome this issue, Zhou (2013) developed humidity indicator using iridescent technology, where nanocrystalline cellulose film was made by casting to form a thick iridescent film and color of these films was adjusted so that they can interact with the electromagnetic field and such films can be explored as a humidity indicator (HI). The dry film appears to be blue-green in color whereas their color shifted to red orange exposing to high humidity. Hong et al. (2010) reported that photonic crystals displayed resistance toward photobleaching and degradation, which makes them a perfect encoding constituent. Binding of target analytes in photonic crystals leads to change in their structural period and eventually shift in their reflection wavelength. Earlier, Tian et al. (2009) using photonic crystal hydrogel concept designed a humidity color indicator through amalgamating the photonic monodispersed latex spheres of poly (styrene-methyl methacrylate-acrylic acid) with photo-polymerized acrylamide. This indicator works reversibly to the presence of relative humidity (RH) i.e., it changes its color from transparent to violet, blue, cyan, green and red as RH changed from 20 to 100%. Zulian et al. (2012) using the similar approach developed an emulsion radical (which is a co-polymerization of styrene and methacrylic acid monomers) and created core-shell latex particles with diverse degrees of order with an array of structural colors.

## Current scenario of nanotechnology application in food packaging

The application of nanoparticles is explored in numerous sectors such as electronics, medicine, textiles, defense, food, agriculture, and cosmetics. Nanotechnology caters several areas of food sciences such as food safety, packaging, processing, bioavailability, fortification, encapsulation, pathogen detection etc. (Weiss et al., 2006; Ravichandran, 2010). Nanotechnology based food packaging offer numerous advantages over conventional food packaging materials *via* improving several properties such as temperature resistance, enhanced durability, flame resistance, barrier, recycling and optical properties, processability due to lower viscosity; proficiently delivery of active materials into the biological systems, at minimum costs with lessen environmental issue. Such progressions make it an ideal candidature for the development of nano materials in wide array of food packaging applications such as processed meat and meat products, cheese, confectionery, cereals, boil-in-the-bag foods, in addition to this it also helps in extrusion-coating applications for fruit juices and dairy products, or co-extrusion processes for the manufacture of bottles for beer and carbonated drinks (Bumbudsanpharoke and Ko, 2015; Trujillo et al., 2016). The application of nanomaterials as food packaging materials has been observed by various authors in several food systems which have been summarized in Table 1.

**Table 1:** Application of nanomaterials as food packaging materials in several food systems (2005–2015).

**Nanomaterial**	**Application of nanomaterial**	**Types of food**	**Effect**	**References**
Oxygen indicator	Nanosensors	Uncooked bacon	Change in sensor color indicate exposure to O_2_	Mills, 2005
Xanthine and hypoxanthine chemical indicator	Nanosensors	Canned tuna	Checked the freshness of food sample	Cubukcu et al., 2007
TiO_2_-coated oriented-polypropylene	Antimicrobial	Lettuce	2 log reduction of *E. coli*	Chawengkijwanich and Hayata, 2008
Carbon nanotubes	Nanosensors	Meat	Detection of pathogens in food	Yang et al., 2008
Absorbent pads containing Ag nanoparticles (NPs)	Antimicrobial	Poultry meat	Effective against *E. coli* and *S. aureus*	Fernandez et al., 2009
Xanthine amperometric sensor	Nanosensor	Fish	Detected the freshness of sample	Shan et al., 2009
Ag montmorillonite NPs	Antimicrobial	Fresh fruit salad	Inhibited the growth of spoilage microorganisms and preserve the sensory quality	Costa et al., 2011
		Fresh cut carrots	Inhibited the growth of spoilage microorganisms and enhanced the shelf life of carrot by more than 2 months stored under 4 ± 1°C	Costa et al., 2012
		Fior di latte cheese	Extended the shelf life up to 3–5 days	Gammariello et al., 2011
Polyvinyl chloride (PVC) with ZnO NPs	Antimicrobial	Sliced apples	Fruit decay rate was significantly lowered	Li X. et al., 2011
Low density polyethylene(LDPE) films loaded with Ag and ZnO NPs	Antimicrobial	Orange juice	Increased the shelf life of orange juice up to 28 days and inactivate *Lactobacillus plantarun*	Emamifar et al., 2011b
Cellulose Ag nanoparticles (AgNPs)	Antimicrobial	Kiwi and melon juices	99.9% reduction of total viable count of bacteria and yeast	Lloret et al., 2012
		Poultry and beef samples	90% reduction of total viable count of lactic acid bacteria	
Carbon nanotubes with allyl isothiocyanate and cellulose	Antimicrobial	Shredded cooked chicken	Inhibited the growth of *Salmonella*	Dias et al., 2013
Low density polyethylene with AgNPs	Antimicrobial	Barberry	2.3 log reduction in molds and 2.84 log reductions of total bacteria	Motlagh et al., 2012
Ethylene vinyl alcohol (EVOH) with AgNPs	Antimicrobial	Chicken, pork, cheese, lettuce, apples, peels, eggshells	2 log reduction of bacterial (*Salmonella* spp., *L*. *monocytogenes*) count in low protein food and 1 log reduction in bacterial count in high protein food	Martinez-Abad et al., 2012
Low density polyethylene with Ag and ZnO	Antimicrobial	Meat	Inhibited the growth of *E. coli, P. aeruginosa* and *L. monocytogenes*	Panea et al., 2013
Pullulan with AgNPs	Antimicrobial	Tsurkey deli meat	Inhibited *L. monocytogenes, S.aureus* over 2 weeks of refrigerated storage	Khalaf et al., 2013
Polyethylene with Ag, TiO_2_	Antimicrobial	Fresh apples, white slice bread, fresh carrots, soft cheese, atmosphere packaging milk powder, fresh orange juice	Inhibited the growth of *Penicillium* and *Lactobacillus* spp.	Metak and Ajaal, 2013
Nanoclays with matrix of polyamide 6	Improved barrier properties	Beef	Enhanced O_2_ barrier properties, capability to block UV and improved stiffness of packaging	Picouet et al., 2014
Sodium alginate with CaCl_2_ and AgNPs	Antimicrobial	Fior di Latte cheese	Increased the shelf life upto 10 days and inhibit the proliferation of *Pseudomonas* spp	Mastromatteo et al., 2015
Isotactic polypropylene (iPP) with CaCO_3_ nanofiller	Antimicrobial	Apple slice	Increased the shelf life up to 10 days	Volpe et al., 2015
Ag/TiO_2_ nanocomposite	Antimicrobial	Bread	Enhanced the shelf life of bread	Cozmuta et al., 2015
Polyethylene with Ag and TiO_2_ NPs	Antimicrobial	Fresh apples, white sliced bread, fresh carrots, pre-packed soft cheese, MAP milk powder and orange juice	Fruit decay rate was significantly lower than in the control sample upto 10 days	Metak, 2015
Polyethylene with Ag, TiO_2_	Antimicrobial	Fresh apples, white slice bread, fresh carrots, soft cheese, atmosphere packaging milk powder, fresh orange juice	Inhibited the growth of *Penicillium* and *Lactobacillus* spp.	Metak and Ajaal, 2013

Nanotechnology in recent years has developed into a wide-ranging, multibillion-dollar global industry. It can be predicted that more nanofood products will come out in the markets within the next few years. Many companies are currently applying nanotechnologies to food could be as high as 400. A number of major food and beverage companies like Altria, Nestle, Kraft, Heinz and Unilever are reported to have curiosity in nanotechnology. Numerous companies are already engaged in production of packaging materials based on nanotechnology that are extending the shelf life of food and drinks and also improving the food safety (Chaudhry et al., 2008; Chaudhry and Castle, 2011; Trujillo et al., 2016).

Nanocor™, a subsidiary of Illinois-based AMCOL International, offers ample range of polymer nanocomposites for purchase in pellet form and packaging products developed with the montmorillonite minerals such as Durethan® KU2-2601 (blending of nylon 6 and nanoclay) has been used as food packaging material *via* improving several properties (gas and moisture barrier, strength, toughness, abrasion, and chemical resistance) of packaging (Duncan, 2011; Cushen et al., 2012; Duran and Marcato, 2013; Bumbudsanpharoke and Ko, 2015). Honeywell International has also developed a mixture of nylon 6-nanoclay composite marketed as Aegis^TM^ OXCE with additional O_2_ scavenger property. It is ideal for packaging of beer and flavored alcoholic beverage and at commercial level; it is flourishing in South Korea (Cooper, 2013; Bumbudsanpharoke and Ko, 2015). In collaboration with Nanocor; Mitsubishi Gas Chemical Company Inc. USA developed nylon nanocomposites with commercial name Imperm® that applied in a barrier layer for multi-layer PET bottle, which is further explored for liquors (beer) andsmall carbonated soft-drink beverages. NanoTuff™(nylon 6 based nanocomposite with 10% clay) is commercialized by Nylon Corporation of America, which exhibit an improved barrier properties to H_2_O, O_2_, and CO_2_, as comparison to neat Nylon 6 (Nanocor, 2008; Duncan, 2011).

Nonosilvers are also engaged in packaging of different food items (fruits, vegetables, herbs, breads, cheeses, soups, sauces, meats etc.) due to their varied antimicrobial properties and globally it is marketed with different commercial names like Fresher Longer^TM^, Bags Fresher Longer^TM^(USA), e.Window® Nano Silver food Container (South Korea); Everin Food Containers Nano Silver Airtight, Incense Nano Silver Food Container, Fresh Box NanoSilver Food Container (South Korea), Zeomic (Japan); Anson Nano, Nano Silver Food (China) (Bumbudsanpharoke and Ko, 2015). This trend is also reflected in the emergence of escalating numeral of national and European R & D projects allied to intelligent packaging (Vanderroost et al., 2014).

## Potential risks, health safety feature and toxicity of nanoparticles in packaging

Current century has observed the speedy development of nanotechnology and its impact in every field therefore for the sake of consumer(s), it is mandatory to have comprehensive information regarding the interface between nanoparticles (NP) and cells, tissues and organisms, particularly in relation to possible hazards to human health. In food science, nanotechnology has an immense impact on food packaging (Chaudhry and Castle, 2011). Nanoparticles may enter into the body through inhalation, ingestion or cutaneous exposure (Maisanaba et al., 2015). Once they penetrate into the biological environment, NP will inevitably come into contact with a huge variety of biomolecules (proteins, sugars, and lipids) which are dissolved in body fluids such as the interstitial fluid between cells, lymph or blood (Farhoodi, 2016). Studies on titania and silver nanoparticles revealed that these materials may enter blood circulation and their insolubility leads to accumulation in organs (Carrero-Sánchez et al., 2006; Kim Y. S. et al., 2008; Rhim et al., 2013). Liver and spleen are mainly responsible for distribution of nanoparticles, mediating their passage from intestine to the blood circulation (Dimitrijevica et al., 2015). A very few studies have taken note of possible toxicity of NP(s) amalgamated in food packaging, besides the data regarding their bioavailability, biodistribution and route followed is scanty. Nanoparticles may unintentionally come in GIT contact *via* leaching/migration of NPs from nanopackaging to food commodities (He and Hwang, 2016). Migration leads to transfer of low molecular mass constituents of packaging material to packaged product. This un-intended transfer of undesirable packaging constituents may raise the safety concerns of consumers. The migration of nanoparticle(s) to food matrix mainly relies on the chemical and physical properties of food and polymer involved. Other controlling parameters includes the concentration, particle size, molecular weight, solubility and diffusivity of specific substance in polymer, pH value, temperature, polymer structure, polymer viscosity, mechanical stress, contact time and composition of food. The solubility of metallic NP in aqueous solution is directly proportional to temperature and inversely proportional to the pH which ultimately augments the migration of metal into the food matrix (Huang et al., 2015). Besides the route of entrance into the body, concentration and duration of exposure, toxicity of nanoparticles also depends upon the host susceptibility and state of organism (Sharma et al., 2009). Aschberger et al. (2011) studied the oral route of transmission and observed that signs of toxicity with relatively high doses of nano-silver or nano-TiO_2_ appeared. Few reports points out toward the genotoxicity and carcinogenicity of NPs. ZnO NP(s) displayed genotoxicity in human epidermal cells, even if the bulk ZnO is non-toxic, implying the role of particle diameter (Sharma et al., 2009). Earlier, Chithrani et al. (2006) reported that the smaller NP(s) exhibit more toxicity than larger ones. High surface area comparative to total mass of smaller NPs enhance their prospects of interaction with the biological molecules, leading to adverse responses. Other factors, such as surface functionalization also plays vital roles (Varela et al., 2012; Shang et al., 2014). Out of few reports, inhalation of very high doses (10 mg/m^3^) of nano-TiO_2_ has been associated with incidence of lung tumors. Inhalation and skin exposure routes are much more explored as compare to ingestion as a route for the entry of NP(s). Inhaled MgO NP(s) can make their way to the olfactory bundle under the forebrain *via* the axons of olfactory nerve in the nose and can also travel to other parts of the brain through systemic inhalation. There are reports documenting the penetration of latex nanoparticles, smaller than 1 μm, through the outer layers of a skin, during constant flexing. In contrast, few other studies question the healthy skin penetration potential of nanostructured particles. Furthermore, inhalation of nanomaterials and probability of their entrance through skin penetration is a matter of high concern, especially for workers and consumers in direct and regular contact (Youssef, 2013). Available data shows that the circulation time increases considerably, in case the NP(s) are hydrophilic and positively charged. Love et al. (2012) also observed that cationic NP seems to be quite toxic than neutral or anionic ones, it might be due to their high affinity toward the negatively charged plasma membrane. Nel et al. (2009) also reported that a cationic NP leads to lysosomal damage and induced cytotoxicity. Nanoparticles that finds their way to bloodstream may influence the blood vessel lining and their function, may lead to blood clotting, or may even contributes toward cardiovascular diseases. Hence, it becomes imperative to obtain data about influence/impact of NP(s) over blood vessels and chances of their crossing the blood brain barrier and migration to the fetus (Dimitrijevica et al., 2015). Avella et al. (2005) showed that tiny amount of particle migration from nanocomposites to foods have been seen during packaging of food and this migration was within the limits prescribed by the European Commission (EC) for silica nanoparticles in clay nanocomposites. Likewise, studies on Ag and ZnO by Panea et al. (2013) also showed that particle migration is within limits as set by EC. Recently, McCracken et al. (2016) also proposed that the size, shape, material, surface charge, solubility and surface chemistry of NP_*S*_ are important in determining their toxicity. Overall data available concludes that more research is warranted before NPs may be tagged as toxic or safe. Forthcoming section briefly discusses the toxicity of some common NP(s) *viz*. SiO_2_, TiO_2_, ZnO, and Ag.

### Silicon oxide nanoparticles

Silica NPs may be toxic through oxidative stress generation, leading to DNA damage and induction of apoptosis. Different scientific reports support this hypothesis in intestinal epithelial cell line models. According to Tarantini and co-workers, silica NPs induced oxidative stress is probably responsible for induction of apoptosis and DNA damage. They exposed Caco-2 cells to silica NP (15 nm) and observed decreased cell viability and increased ROS production at 32 μg/ml dosage with over-expression of caspase-3 at 64 μg/ml. Silica NPs were genotoxic to cells and augmented the frequency of micronucleus formation (Tarantini et al., 2015). As reported by Yang et al. (2014), 10–50 nm silica NPs induced LDH release, indicating reduced cell viability; partial inhibition of cell proliferation and a slight S phase cell cycle arrest in human gastric epithelial cells (GES-1) cells, and S and G2/M phase arrest in Caco-2 cells with minor increase in ROS generation. Overall they didn't report any apoptosis or necrosis, indicated that the ROS generation may accompany decreased viability and cell cycle arrest.

In contrast to the above reports, Moos et al. (2011) reported zero toxicity of silica NPs in intestinal epithelial cells. Caco-2 and colon carcinoma RKO cells showed minimal toxicity induction by silica NPs up to the dosage of 100 μg/cm^2^. Cells exposed to 50 μg/cm^2^ silica NP for 4 hr also showed minimal changes in gene expression as determined by whole genome microarray analysis. These results are further supported by Schubbe et al. (2012), who also observed no cellular toxicity in undifferentiated Caco-2 cells when co-cultured with 32 and 83 nm fluorescent silica NP(s). No cytotoxicity or genotoxicity was observed in cells treated up to 200 μg/ml of NPs. It has also been found that the SiO_2_ NPs can also induce allergen-specific Th2-type allergic immune responses, as observed in an *in vivo* study involving female BALB/c mice exposed to NPs. Intranasal exposure to ovalbumin (OVA) and SiO_2_ NPs induces a relatively high level of OVA-specific immunoglobulin IgE, IgG, and IgG1 antibodies (Yoshida et al., 2011). From the above reports, it can be concluded that the harmful effects of silica NPs are associated with high dosage (i.e., 200 μg/ml) however; the same at a dose of 100 μg/ml are reported safe.

### Titanium dioxide nanoparticles

TiO_2_ NPs have been associated with cytotoxicity mediated through oxidative stress-dependent pathways leading to DNA damage, cell cycle arrest or delay and mitochondrial dysfunction, particularly in pulmonary and inhalation models (Shi et al., 2013). However, experimental data observed in intestinal epithelial cells are in agreement that TiO_2_ NPs are nontoxic. Koeneman et al. (2010) observed that the treatment of Caco-2 cells with TiO_2_ NPs (<40 nm) displayed a decrease in epithelial monolayer integrity by decreased TEER measurements and a loss of localization of γ-catenin to cell adherens junctions, beginning at 6 days after continuous TiO_2_ NP treatment and long-lasting to 10 days at dose of 1,000 μg/ml. No decrease in TEER was examined after acute exposure and no induction of cell death was reported after acute or chronic exposure. Chalew and Schwab (2013) observed the toxicity of P25 TiO_2_ (25% rutile and 75% anatase) treatment (100 μg/ml) on the intestinal epithelial cell lines, Caco-2 and SW480. No toxicity in Caco-2 cells was observed. Few studies had supported that TiO_2_ NP can disrupt normal microvilli structure in intestinal epithelial cells, which affects the normal cellular functions, particularly nutrient absorption (McCracken et al., 2016).

### Zinc oxide nanoparticles

As reported earlier, ZnO NPs may lead to toxicity due to of the NP dissolution either in outside or within the cells, leading to enhanced availability of zinc ions, which interacts with enzymes and other cell components; oxidative stress and lysosomal destabilization; and mitochondrial dysfunction contributing to the cytotoxic response (Vandebriel and De Jong, 2012). Song et al. (2014) observed that 90 nm ZnO NPs at a concentration of 10 μg/ml decreases Caco-2 cell viability, inhibits cell proliferation, enhance ROS generation along with SOD levels; signifying an oxidative stress response. In another study, MTT and LDH assays revealed the dose-dependent toxicity of ZnO NPs in Caco-2 cells (Kang et al., 2013).

### Silver nanoparticles

Silver nanoparticles are genotoxic, cytotoxic and even carcinogenic. The nano size of NPs allows them to cross the cellular barrier, leading to the formation of free radicals in the tissues and eventually leading to oxidative damage to the cells and tissues (Pradhan et al., 2015). In several *in vitro* studies, Ag NPs displayed toxicity through an oxidative stress-dependent mechanism as well as through oxidative stress-independent intracellular effects. It has been observed that the exposure of human lung fibroblasts and glioblastoma cells to 6–20 nm Ag NPs increased ROS production, induced mitochondrial injury and DNA damage and also induced G2/M phase cell cycle arrest (Rani et al., 2009). Similar toxicity has been observed in intestinal epithelial cells (Bohmert et al., 2012). In another study, Aueviriyavit et al. (2014) observed that <100 nm Ag NPs can be internalized by cells with a dose-dependent decrease in cell viability, starting at 10 μg/ml. Treatment with Ag NPs also induced activation of the stress-responsive gene Nrf2 and heme oxygenase-1 (HO-1). Recently, McCracken et al. (2016) also demonstrated that Ag NP-induced oxidative stress responses in intestinal epithelial cells. However, Song et al. (2014) reported that co-incubation of Caco-2 cells with 10 μg/ml of 90 nm Ag NPs decreased the cell activity, but no induction of cell death was observed. In another study, Kumar (2015) reported that Ag NPs caused depolarization of α-tubulin, a major component of microtubule, having adverse effect over the cellular structure and associated cytoskeleton of the cell.

Other nanomaterial(s), such as carbon NPs are also known to cause allergic inflammation and it has been reported that the single and multi-walled carbon nanotubes increased lung inflammation and allergen-specific IgE levels in mice sensitized to OVA egg allergen. In another study, multi-walled carbon nanotubes with preexisting inflammation increased airway fibrosis in mice with allergic asthma (He and Hwang, 2016).

Besides these concerns, application of some nanocomposites triggered concerns regarding their environmental impacts, due to their non-biodegradable nature. Therefore, eco-toxicity studies on nanoparticles are mandatory before their commercial applications. Overall, the existing reports regarding the toxicity of NPs are not in full agreement with each other and also not fully conclusive. There are ambiguous results leading to a vague situation regarding the toxicity of NP to human beings. Some reports indicate total metal migration, over others showing particle migration. An imperative conclusion from the above discussion is that if nanoparticles are completely covered or encapsulated by the host polymer matrix, then the probability of migration into food matrix is quite less. However, during un-intentional mechanical impact on the food contact surface, could alter the smooth properties or in case of cut edges or technically improperly manufactured polymer nanocomposite with nanoparticles may lead to its release. In lieu of the above, manufacturers should reassure fully incorporation of nanoparticles in films or molded articles (Stormer et al., 2017). Nevertheless, the available scientific data on toxicity or migration of NPs is still at infancy stage and additional meticulous analysis is required before their vast application. The ultimate fate and toxicity of nanomaterials in food packaging depend on the physiochemical characteristics and dosage. Safe application of nanotechnology to the food packaging requires systematic characterization and assessment *in silico, in vitro*, and *in vivo*. Altogether, taking into thought, a varied number of physical, chemical and biological factors, their absorption, distribution, metabolism, excretion and lastly their toxicity should be quantified and evaluated for risk assessment to consumers (He and Hwang, 2016).

## Concluding remarks

The quality of food items chiefly rely over their perishability. Perishable foods require ambient temperature to maintain quality and freshness during transit and storage. Monitoring the extent to which perishable foods encounter degradation promoting factors chiefly, oxygen, light and ethylene can control perishability. Food package and the packaging material involved play an important and decisive role in food quality and shelf life. Packaging chiefly influences the barrier properties to form an irrefutable food environment. The dawn of nanotechnology has further opened up new avenues and technological advancement possibilities in food packaging area. Linking of nanoparticles to polymer to fabricate nanomaterial packaging potentiates routine packaging with enhanced barrier properties, mechanical and thermal strength, flexibility and stability. Time-consuming quality-control analysis as well as consumer technical illiteracy is another key problem surfacing the food industry. Novel nano-packaging systems (Improved/Active/Intelligent packaging) have potential to serve as an important tool to overcome existing packaging challenges with consumer and industrialist satisfaction. It is anticipated that conventional packaging will be thoroughly replaced with multifunctional smart or active packaging. Nano-structured materials put a check to microbial invasion, assuring microbial food safety. Additionally, nanosensors alert and warn consumers regarding the safety and accurate nutritional status of the packaged food. Several corporations have entered in this area with introduction of new packaging systems with updated technology. However, being a young branch, gaps in knowledge exists, leaving ample questions to the scientific community; mainly concerning its toxicity and ecotoxicity. Concerns regarding nanoparticles migration to packaged foodstuffs has been raised, however, migration assays and risk assessment are still not conclusive. Undefined toxicity, scarcity of supportive clinical trials data and risk assessment studies limits the application of nanomaterial in the food packaging sector. Lastly, for the successful implementation of nanotechnologies at gigantic scale, consumer's approval is mandatory. Both benefits and risks assessment should be acknowledged undoubtedly. Accredited research bodies should come forward with appropriate labeling and set down common regulations that boost the consumer acceptability. Government agencies should come forward and work in co-ordinance with each other to tackle this issue sensibly and gallantly and make a way for developing world. Legislation and strategies should be framed prudently for the sake of civilization concerning the management and application of nanomaterial in food packaging systems.

## Author contributions

CS and RD have contributed equally to this review work. NR helped in updating the manuscript. HP assisted in compilation and proper editing of this review.

### Conflict of interest statement

The authors declare that the research was conducted in the absence of any commercial or financial relationships that could be construed as a potential conflict of interest. The reviewer FP and handling Editor declared their shared affiliation, and the handling Editor states that the process nevertheless met the standards of a fair and objective review.
